# P02.180. Arts speech therapy influences heart rate variability indices

**DOI:** 10.1186/1472-6882-12-S1-P236

**Published:** 2012-06-12

**Authors:** U Wolf, U Gerber, M Wolf, S Klein, F Scholkmann

**Affiliations:** 1 University of Bern, Institute of Complementary Medicine KIKOM , Bern, Switzerland; 2 University Hospital Zurich, Zurich, Switzerland

## Purpose

Previous studies revealed that arts speech therapy (AST) affects the cardiorespiratory system, hemodynamics and oxygenation. The aim of this study was to further investigate effects of AST by assessing changes in heart rate variability (HRV) indices.

## Methods

Measurement in 24 adults comprised 8 minutes pre-baseline, 5 minutes recitation, 5 minutes recovery, 5 minutes recitation, and 20 minutes post-baseline. Measurements were performed for 3 different AST tasks [recitation of alliterative (RA), hexameter (RH), and prose (RP) verses] and a control task [mental arithmetic (MA) with voicing of the result] in a randomized crossover design. HRV was determined using a Medilog AR12 holter ECG. Multifractality of HRV was determined using the multifractal detrending moving average method. Statistical analysis was applied to the difference between pre-baseline, 2 recitation and 5 baseline periods. The four tasks were tested separately; p≤0.05 was considered significant.

## Results

(1) During recitation: Heart rate increased during RA, RH and MA but not during RP. The coefficient of variation increased during RH and MA. Normalized high frequency (nHF, 0.15-0.4 Hz) and low frequency (nLF, 0.04-0.15 Hz) power of the HRV decreased (nHF) and increased (nLF) during RA, RH and RP. The multifractal parameter αmode showed a decrease during MA. The degree of multifractality (Δα) increased during PR and MA. Heart rate coherence (HRC) decreased during RP, RH and MA. (2) After recitation: PR caused an increase in Δα; RH caused an increase in the very low frequency (VLF, <0.04 Hz) power, and the HR decreased after RA and RH. HRC decreased after RH.

## Conclusion

AST affects HRV indices during and after the AST. The changes indicate that AST during the recitation decreases the activity of the parasympathicus. The multifractality of HRV changed during MA and after MA and PR. The decrease in HRC after RH indicates a change in the activity of the hypothalamus-pituitary-adrenal axis.

